# Synergistic Toughening Mechanisms in ZrO_2_/Multi-Walled Carbon Nanotubes-Reinforced CaZr_4_(PO_4_)_6_ Ceramics for Enhanced Mechanical Performance

**DOI:** 10.3390/ma18102289

**Published:** 2025-05-14

**Authors:** Junyao Shen, Tian Si, Huan Gao, Linhua Zhu, Heng Zhang, Xin Gao, Xiaoning Tang

**Affiliations:** School of Chemical Engineering, Kunming University of Science and Technology, Kunming 650504, China

**Keywords:** CZP ceramics, ZrO_2_ and MWCNTs, mechanical properties, toughening mechanisms

## Abstract

ZrO_2_ and multi-walled carbon nanotubes (MWCNTs) were selected as single-phase and composite toughening agents to investigate the influence on the mechanical properties of CaZr_4_(PO_4_)_6_ (CZP) ceramics. The results revealed that the addition of single-phase or composite toughening agents had minimal impact on the phase composition and crystallinity of CZP ceramics. When the content of the single-phase ZrO_2_ toughening agent reached 10 wt.%, the flexural strength of CZP ceramics increased to 71.60 MPa due to the particle toughening mechanism of ZrO_2_. With the addition of 1.0 wt.% ZrO_2_ and 0.3 wt.% MWCNTs, the CZP ceramics demonstrated enhanced densification and improved sintering activity. The small-sized ZrO_2_ particles were evenly dispersed within the ceramic matrix, accompanied by a phase transformation during sintering. Together with MWCNTs, this combination resulted in a significant increase in flexural strength, reaching 138.43 MPa. An in-depth analysis of the toughening mechanisms indicated that the CZP ceramic matrix primarily featured ZrO_2_ phase transformation toughening and the pull-out and bridging toughening provided by MWCNTs. The synergistic interaction of these multiple toughening mechanisms significantly enhanced the mechanical properties of CZP ceramics, providing valuable theoretical insights for optimizing the performance of phosphate ceramics in practical applications.

## 1. Introduction

Calcium zirconium phosphate-CaZr_4_(PO_4_)_6_ (CZP) belongs to the Sodium Zirconium Phosphate-NaZr_2_(PO_4_)_3_ (NZP) family of ceramic materials and has attracted considerable interest due to its distinctive properties [1,2,3]. Recent research has focused on the low thermal expansion characteristics of NZP ceramics and their applications in materials designed for thermal shock resistance [4,5,6]. However, NZP ceramics frequently encounter challenges related to poor sintering performance and the formation of micro-cracks, which can significantly reduce their mechanical strength and overall effectiveness under extreme conditions.

Previous studies have attempted to enhance the mechanical strength of NZP ceramics through optimizing sintering conditions and substituting various ions. Liu et al. [7] fabricated CZP ceramics with reduced grain size and lower porosity via fast hot-pressing sintering, elevating the flexural strength of CZP ceramics to 94.5 MPa. Wang et al. [8] synthesized Ca_0.5_Sr_0.5_Zr_4−x_Ti_x_P_6_O_24_ (x = 0–0.2), substituting Zr^4+^ with Ti^4+^, which improved the ceramic flexural strength to 66.5 MPa. Kumar et al. [9] prepared ZnCZP ceramics by partially replacing Ca^2+^ with Zn^2+^, achieving a 150% increase in the flexural strength of CZP ceramics. However, the enhancement of NZP ceramic toughness through these improved sintering conditions and ion-substitution techniques has revealed certain limitations. Consequently, it is essential to investigate alternative methodologies for the effective toughening of ceramics [10,11,12].

In the field of ceramic toughening, the incorporation of secondary phases like zirconium dioxide (ZrO_2_) is a promising strategy [13,14,15] due to the effectiveness of this oxide to enhance particle toughening and phase transformation mechanisms, which can substantially improve ceramic mechanical properties. Elsaka et al. [16] used it to toughen lithium silicate glass-ceramic, improving the fracture toughness, flexural strength, elastic modulus, and hardness. Zheng et al. [17] synthesized Al_2_O_3_/ZrO_2_ eutectic ceramics under ultra-high temperature, with the ceramic’s fracture toughness reaching a value of 10.6 MPa·m^1/2^ and a Vickers hardness of 17.8 GPa, highlighting the t-ZrO_2_ phase-transformation toughening. Li et al. [18] toughened Ti(C,N) ceramics with 3Y-ZrO_2_ and WC, indicating that the addition of 15 wt.% 3Y-ZrO_2_ and 15 wt.% micro-WC resulted in optimal mechanical properties (flexural strength, fracture toughness, and Vickers hardness of 1501 MPa, 7.25 MPa·m^1/2^, and 19.97 GPa, respectively).

Multi-walled carbon nanotubes (MWCNTs) are effective second-phase toughening agents [19,20,21,22], exhibiting remarkable mechanical properties that enable exceptional toughening effects. Liao et al. [23] toughened Si_2_BC_3_N ceramics with MWCNTs. Si_2_BC_3_N ceramics with 1 vol.% MWCNTs addition had higher flexural strength (462.1 MPa) and fracture toughness (5.54 MPa·m^1/2^). Akinribide et al. [24] prepared TiN-MWCNTs composite ceramics with 1 wt.% MWCNTs content, and these exhibited the highest fracture toughness and Vickers hardness, which were 12.22 MPa·m^1/2^ and 40 GPa, respectively. In our previous research, Gao et al. [25] used MWCNTs to toughen CZP ceramics, increasing their flexural strength (173%) and flexural modulus (156%).

Furthermore, using multiple toughening agents in composite materials is an effective way to enhance ceramic properties [26,27]. This approach capitalizes on the synergistic effects of different toughening mechanisms for mechanical performance. Zhu et al. [28] toughened Al_2_O_3_ ceramics with 3.0 wt.% SiC particles and 1.0 wt.% SiC whiskers, increasing Vickers hardness to 18.8 GPa (a 9.3% rise) and fracture toughness to 4.8 MPa·m^1/2^ (a 29.7% increase). Shi et al. [29] toughened Al_2_O_3_ ceramics with B_4_C@TiB_2_; the Vickers hardness and fracture toughness of the toughened ceramics were measured as 21.5 GPa and 6.92 MPa·m^1/2^, respectively. Cui et al. [30] toughened Al_2_O_3_/Ti(C,N) ceramics with 0.4 wt.% GNPs and 1.0 wt.% ZrO_2_, achieving a fracture toughness exceeding 11 MPa·m^1/2^, about 86% higher than the untoughened ceramic.

While individual toughening agents such as ZrO_2_ and MWCNTs have demonstrated their effectiveness, the continued exploration of composite toughening methodologies may lead to further advancements in the mechanical properties of ceramic materials like CZP, paving the way for broader applications in demanding environments. In our research, ZrO_2_ and MWCNTs were utilized as composite toughening agents to enhance the toughness of CZP ceramics, improving their mechanical properties. The synthesis of CZP ceramic powders was accomplished through the coprecipitation method. Adding the toughening agents into the ceramic powders was achieved through multiple ultrasonic treatments, significantly enhancing the bonding strength between the toughening agents and the ceramic matrix. A series of ZrO_2_/MWCNTs/CZP composite ceramics were fabricated with varying concentrations of the toughening agents. Subsequently, a comprehensive analysis was conducted to investigate the crystal structure, microstructure, mechanical properties, thermal expansion characteristics, and toughening mechanisms associated with the composite materials.

## 2. Experimental

### 2.1. Materials Preparation

The chemical reagents used in the experiment are shown in Table 1.

The preparation of CZP ceramic powder and the pretreatment of multi-walled carbon nanotubes (MWCNTs) are based on our previously published research [25]. The toughening agents, ZrO_2_ and pretreatment MWCNTs, are incorporated using a post-addition method to improve the mechanical properties of the composite materials.

The preparation process is as follows: the CZP ceramic powder and toughening agents are separately immersed in ethanol solutions and subjected to ultrasonic treatment for 10 min. Following this treatment, the ultrasonically processed suspensions are thoroughly combined, and an appropriate quantity of the sintering agent MgO (4 wt.%) is added. After ultrasonic treatment for 10 min, a stable suspension is achieved. The mixture is transferred to a planetary ball mill for ball-milling treatment (400 rpm, 8 h), and the composite ceramic powder A/CZP (with A representing the toughening agent) is obtained.

**Figure 1:**
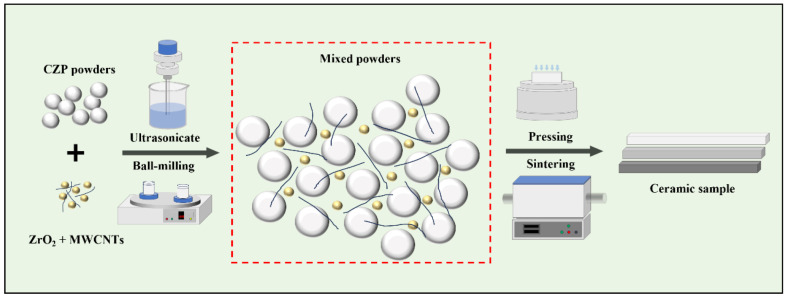
The preparation process of ZrO_2_/MWCNTs/CaZr_4_(PO_4_)_6_ ceramic samples.

Subsequently, the composite ceramic powder is dry-pressed into a green body with dimensions of 5 mm × 5 mm × 50 mm, under a pressure of 20 MPa using a dry-pressing molding process. This green body is placed in a high-temperature sintering furnace and sintered in an air or argon atmosphere. The temperature is gradually raised to 1100 °C at a consistent heating rate of 5 °C/min and maintained for 2 h. After cooling to room temperature, the composite ceramic samples are designated as x/y wt.% A/CZP, where x and y are the dosages of the toughening agents (with x = 1, 5, 10, 15 and y = 0.1, 0.3, 0.5, 0.7). The detailed preparation process is illustrated in Figure 1.

### 2.2. Characterization Methods

X-ray diffraction is performed using X’Pert3 Powder made by PANalytical in Almelo, The Netherlands for detection with Cu-Kα radiation (2θ range of 10–90°). SEM was used to observe the microstructure of the sample using ZEISS Sigma 300 made in Jena, Germany. The Archimedes method was used to measure the bulk density of ceramic samples. The coefficient of thermal expansion is tested using DIL402 Expedis Classic made by Netzsch in Selb, Germany with a heating rate of 5 °C/min from 30 to 1000 °C. An electromechanical universal testing machine ETM-103A made by WANCE in Shenzhen, China is used to perform the four-point bending test (The results of density and bending tests are taken as the average of 5 samples).

The calculation of flexural strength and flexural modulus is shown in the following formula [25]:

(1) $$\left(\delta_{f}\right)=\frac{3Fa}{bd^{2}}$$

(2) $$\left(E_{f}\right)=\frac{23L^{3}F}{108bd^{3}∆f}$$

where δf is the flexural strength (MPa), Ef is the flexural modulus (GPa), F is maximum load (N), a is the distance between upper and lower rollers (1/4 span length, 10 mm), b is the width of specimen (mm), d is the height of specimen (mm), L is the span length (mm), and Δf is the displacement (mm).

## 3. Results and Discussion

### 3.1. ZrO_2_ Single-Phase Toughening CZP Ceramics

In this study, ZrO_2_ was selected as a single-phase toughening agent, and the influence of ZrO_2_ content on the phase composition, microstructure, mechanical properties, and toughening mechanism of CZP ceramics were thoroughly investigated.

**Figure 2:**
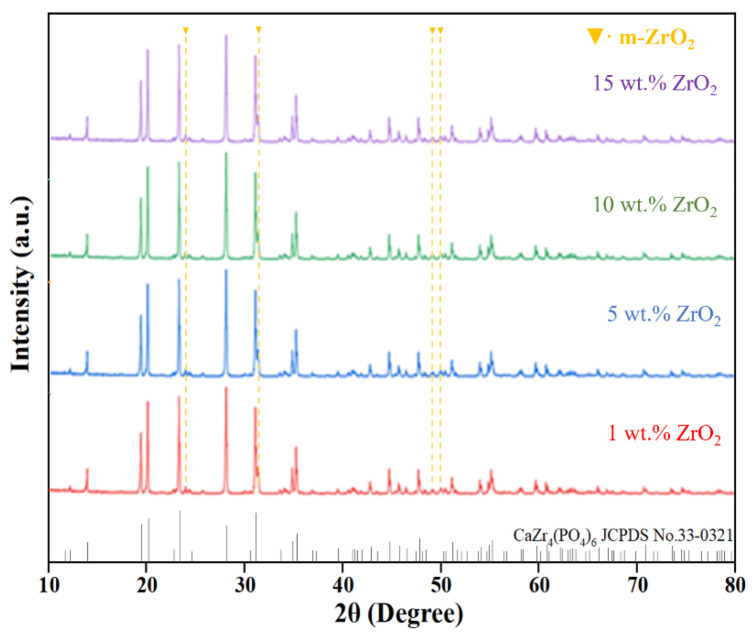
XRD patterns of ZrO_2_/CaZr_4_(PO_4_)_6_ ceramics.

Figure 2 illustrates the XRD patterns of ZrO_2_/CZP (Z/C) ceramics with different amounts of ZrO_2_ added. A comparison with the standard card (JCPDS No: 33-0321) reveals that all diffraction peaks in the samples are sharp, indicating that CZP achieves complete crystallization under the specified preparation conditions. Notably, the addition of ZrO_2_ did not affect the phase of CZP. Furthermore, the characteristic diffraction peaks of monoclinic ZrO_2_ (m-ZrO_2_) were identified in the patterns, with no other ZrO_2_ phases observed.

**Figure 3:**
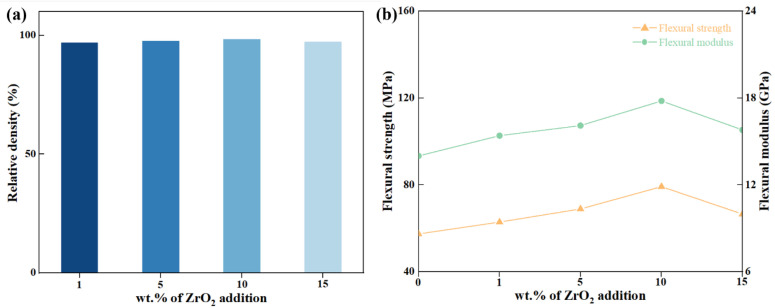
Mechanical properties of ZrO_2_/CaZr_4_(PO_4_)_6_ ceramics: (**a**) relative density; (**b**) flexural strength and flexural modulus.

The analysis of the relative density variation results of Z/C ceramic samples (as illustrated in Figure 3a) revealed that adding ZrO_2_ had a relatively minor impact on the bulk density of the CZP ceramics. A maximum density of 3.22 g/cm^3^ was achieved with a 10 wt.% addition of ZrO_2_. The results of the mechanical property tests for the Z/C ceramic samples are presented in Figure 3b. The figure illustrates that, with increasing amounts of ZrO_2_, both the flexural strength and flexural modulus initially increased before decreasing. This trend was consistent with the changes observed in relative density. Specifically, the 10 wt.% Z/C ceramic sample reached a maximum flexural strength of 71.60 MPa, reflecting a 137% increase compared to pure-phase CZP ceramics. These results demonstrate that incorporating ZrO_2_ can effectively enhance the mechanical properties of CZP ceramics. Detailed experimental data are provided in Table 2.

**Figure 4:**
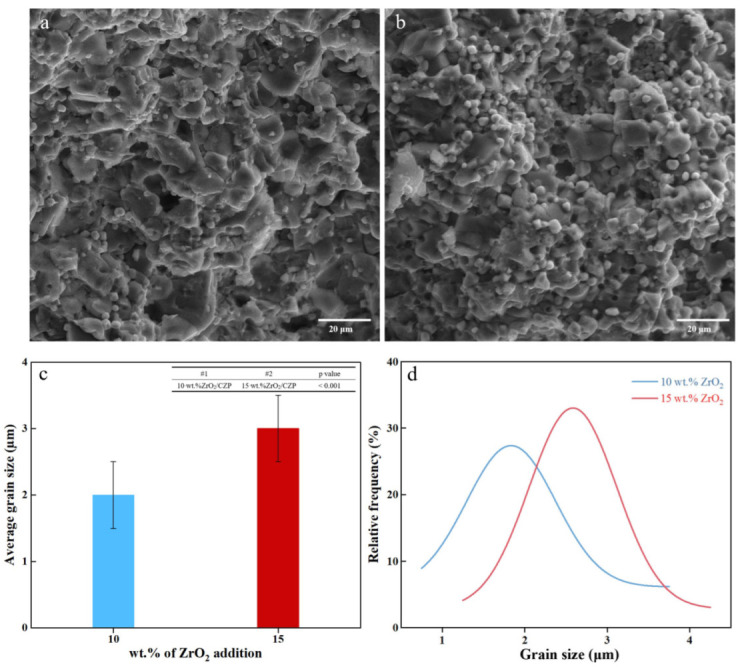
SEM micrographs of ZrO_2_/CaZr_4_(PO_4_)_6_ ceramics: (**a**) 10 wt.% ZrO_2_/CaZr_4_(PO_4_)_6_; (**b**) 15 wt.% ZrO_2_/CaZr_4_(PO_4_)_6_; (**c**) average particle size, and (**d**) particle size distribution.

To investigate the enhancement mechanism of ZrO_2_ on the mechanical properties of CZP ceramics, scanning electron microscopy (SEM) was employed to analyze the particle size and dispersion of the toughening agent ZrO_2_ within the ceramic matrix. Given that the particle size distributions of ZrO_2_ and mechanical properties of the 1 wt.% and 5 wt.% Z/C ceramic samples show negligible differences compared with those of CZP ceramics, the 10 wt.% Z/C ceramic sample, which demonstrates the optimal flexural strength, was chosen as the primary sample of this study. Meanwhile, the 15 wt.% Z/C ceramic sample was chosen as a comparative. This experimental design aimed to comprehensively explore the impact of changes in ZrO_2_ particle size and distribution on the mechanical properties of CZP ceramics. The results are depicted in Figure 4.

As shown in Figure 4a, the CZP ceramics feature relatively large particle sizes, ranging from 6 to 9 µm, with a notable presence of pores between the particles. Upon adding 10 wt.% ZrO_2_, a substantial number of ZrO_2_ particles appear on the surfaces of the CZP particles, concentrated around 2 µm. These ZrO_2_ particles exhibit excellent dispersion and strong bonding with the CZP particles. However, when the ZrO_2_ content is increased to 15 wt.%, there is a significant increase in the particle size of ZrO_2_, with sizes focused around 3 µm. This results in noticeable agglomeration, reducing the degree of bonding with the ceramic matrix.

Based on a review of the relevant literature, the toughening mechanisms of ZrO_2_ in ceramics mainly involve particle and transformation toughening. The XRD results indicate that only m-ZrO_2_ exists in the Z/C ceramic samples. This implies that the enhancement of mechanical properties in CZP ceramics largely arises from ZrO_2_ particle toughening. The principle of ZrO_2_ particle toughening is mainly reflected in micro-crack deflection and stress dissipation. Therefore, factors such as particle size, distribution, and the amount of ZrO_2_ added are of particular importance.

The ZrO_2_ particles, being smaller in size, are uniformly dispersed within the ceramic matrix, creating more sites for toughening. When external forces are applied to the ceramic and cracks develop, this leads to phenomena such as crack deflection and bridging during the propagation of the cracks. SEM images reveal that the Z/C ceramics with a 10 wt.% ZrO_2_ addition exhibit superior particle size and dispersion compared to 15 wt.% Z/C, offering distinct advantages in enhancing mechanical properties. The strong bonding between ZrO_2_ and the CZP ceramic matrix allows the ZrO_2_ to detach from the matrix surface during fracture, thus distributing and dissipating the internal stress of the material. This process effectively hinders the further propagation of cracks, contributing to the toughening effect.

In summary, when the amount of ZrO_2_ added is 10 wt.%, the CZP ceramics exhibit high density and crystallinity. The fine particle size and effective dispersion of ZrO_2_ allow the ceramic to utilize its particle toughening mechanism efficiently during stress-bearing processes, effectively impeding crack propagation and resulting in a marked improvement in mechanical properties.

### 3.2. ZrO_2_ and MWCNTs Composite Toughening CZP Ceramics

In previous work, we selected multi-walled carbon nanotubes (MWCNTs) as a single-phase toughening agent to enhance the mechanical strength of CZP ceramics. The findings revealed that MWCNTs had a remarkable toughening effect on CZP. When the addition of MWCNTs reached 0.5 wt.%, the flexural strength of CZP ceramics was as high as 91.76 MPa [25]. Although both ZrO_2_ and MWCNTs improved the flexural strength of CZP ceramics as single-phase toughening agents, the enhancement was still relatively limited. To optimize the mechanical properties, we attempted to select ZrO_2_ and MWCNTs as composite toughening agents and explore their influence on the mechanical properties of ceramics and the underlying mechanism to develop CZP ceramics with superior mechanical properties.

#### 3.2.1. Phase Analysis

**Figure 5:**
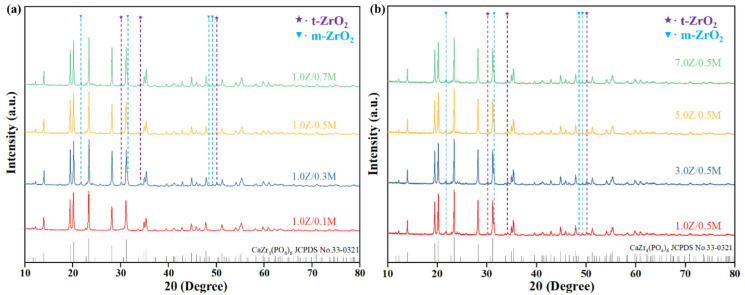
XRD patterns of ZrO_2_/MWCNTs/CaZr_4_(PO_4_)_6_ ceramics: (**a**) 1.0 wt.%ZrO_2_/x wt.% MWCNTs/CaZr_4_(PO_4_)_6_ ceramics; (**b**) x wt.%ZrO_2_/0.5 wt.% MWCNTs/CaZr_4_(PO_4_)_6_ ceramics.

Figure 5 illustrates the XRD patterns of ZrO_2_/MWCNTs/CZP (Z/M/C) ceramic samples prepared with varying contents of ZrO_2_/MWCNTs composite toughening agents. The patterns reveal sharp and well-defined diffraction peaks, indicating that all the CZP ceramics manufactured under these conditions possess good crystallinity. This observation confirms that the addition of the composite toughening agent does not significantly impact the phase of CZP. Furthermore, monoclinic ZrO_2_ (m-ZrO_2_) and tetragonal ZrO_2_ (t-ZrO_2_) were detected in the patterns of all ceramic samples. Unlike the case of single-phase ZrO_2_ toughening, the presence of MWCNTs led to a phase transformation of ZrO_2_ during the sintering process of the CZP ceramics. Specifically, some m-ZrO_2_ converted to t-ZrO_2_ and remained stably incorporated within the CZP ceramic matrix.

#### 3.2.2. Mechanical Properties

**Figure 6:**
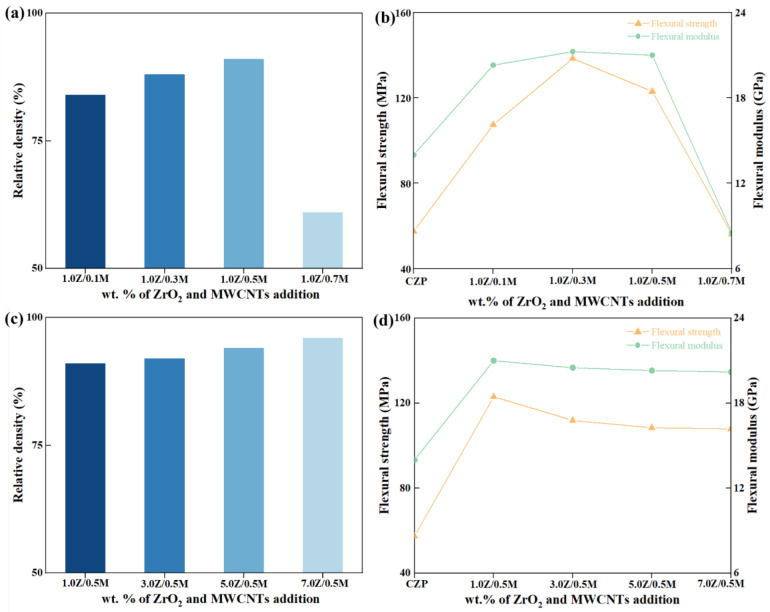
Mechanical properties of ZrO_2_/MWCNTs/CaZr_4_(PO_4_)_6_ ceramics: (**a**,**c**) relative density, and (**b**,**d**) flexural strength and flexural modulus.

Figure 6 illustrates the variations in relative density, flexural strength, and flexural modulus of CZP ceramics with the addition of the composite toughening agent ZrO_2_/MWCNTs. As depicted in Figure 6a,c, when the amount of ZrO_2_ is maintained at 1.0 wt.%, the relative density of CZP ceramics exhibits a gradual increase as the quantity of MWCNTs rises. However, when the MWCNTs content reaches 0.7 wt.%, there is a sharp decline in the density of the CZP ceramics. This drop may be due to significant agglomeration of MWCNTs during the sintering process, which increases the internal defects within the CZP ceramics. When the MWCNTs content is set at 0.5 wt.%, the relative density of the CZP ceramics shows a slight increase with the addition of ZrO_2_. This improvement is likely attributed to the ability of small-sized ZrO_2_ particles to fill the gaps or pores within the ceramic matrix, thereby improving its density.

As illustrated in Figure 6b,d, following the introduction of the composite toughening agent, the changes in the flexural strength and flexural modulus of CZP ceramics closely mirror the trends observed in relative density. In particular, with ZrO_2_ content fixed at 1.0 wt.% and MWCNTs at 0.3 wt.% (resulting in the 1.0Z/0.3M/C sample), the maximum flexural strength achieved is 138.43 MPa. This represents a 240% increase compared to the CZP ceramic matrix without the toughening agent. These results clearly indicate that the CZP ceramic matrix enhanced with the composite toughening agent ZrO_2_/MWCNTs exhibits significantly improved mechanical properties. The detailed experimental data are presented in Table 3.

#### 3.2.3. Morphological Analysis

**Figure 7:**
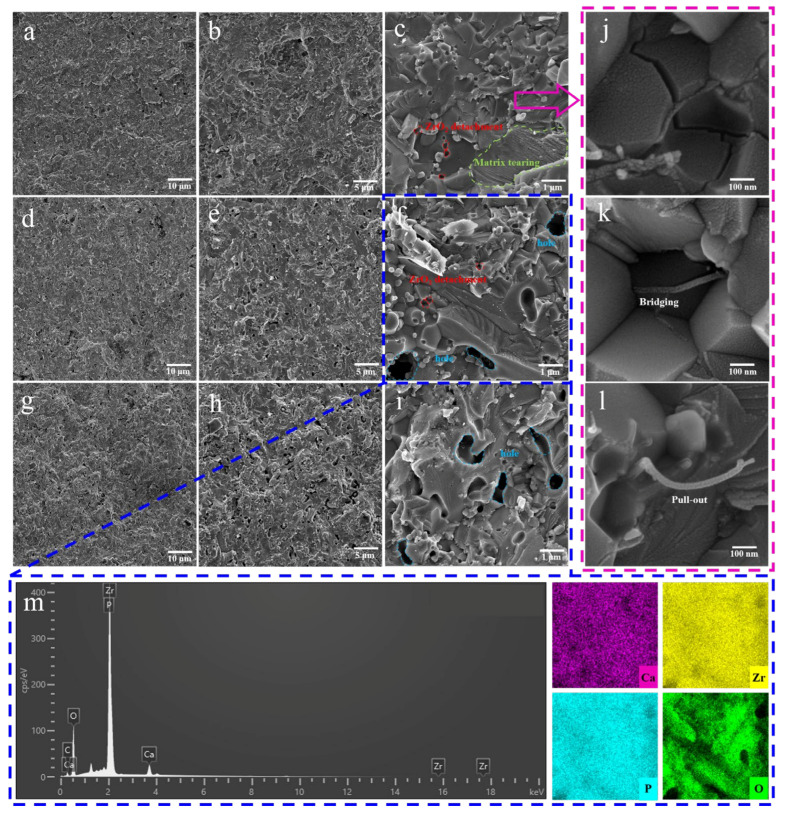
SEM micrographs of ZrO_2_/MWCNTs/CaZr_4_(PO_4_)_6_ ceramics: (**a**–**c**) 1.0 wt.% ZrO_2_/0.3 wt.% MWCNTs/CaZr_4_(PO_4_)_6_; (**d**–**f**) 1.0 wt.% ZrO_2_/0.5 wt.% MWCNTs/CaZr_4_(PO_4_)_6_; (**g**–**i**) 3.0 wt.% ZrO_2_/0.3 wt.% MWCNTs/CaZr_4_(PO_4_)_6_; (**j**–**l**) MWCNTs in 1.0 wt.% ZrO_2_/0.3 wt.% MWCNTs/CaZr_4_(PO_4_)_6_; and (**m**) EDS results of 1.0 wt.% ZrO_2_/0.5 wt.% MWCNTs/CaZr_4_(PO_4_)_6_.

To conduct a comprehensive exploration of the existence state and toughening mechanism of the composite toughening agent ZrO_2_/MWCNTs in CZP ceramics, a SEM analysis was performed on the cross-section of the ceramic body. As shown in Figure 7a–c, the 1.0Z/0.3M/C ceramic sample demonstrates minimal pores and gaps, exhibiting a uniform surface and remarkable compactness. The ZrO_2_ particles are well-dispersed within the matrix, displaying relatively uniform particle sizes, and distinct tearing traces are visible. Meanwhile, Figure 7j–l clearly illustrates the presence of MWCNTs, highlighting notable pull-out and bridging phenomena.

When CZP ceramics fracture under external forces, ZrO_2_ particles on the surface, which are firmly bonded to the matrix, absorb a certain stress during separation from the ceramic matrix, contributing to an enhancement in the flexural strength of CZP. The numerous tearing traces observed on the ceramic matrix indicate strong inter-granular bonding, further evidencing the high strength. Moreover, when CZP ceramics are subjected to external forces, they can initiate a phase transformation from t-ZrO_2_ to m-ZrO_2_, as shown in Figure 5a. The volume expansion and shear strain from phase transformation can offset part of external stress, prevent crack propagation, and improve the mechanical strength. Previous research has confirmed that the primary toughening mechanisms for MWCNTs in CZP ceramics are bridging and pull-out [25]. The findings reveal that CZP ceramics with the addition of 1.0 wt.% ZrO_2_ and 0.3 wt.% MWCNT achieve the highest surface densification and optimal sintering activity. Additionally, the toughening mechanisms of phase transformation in ZrO_2_, together with the bridging and pull-out effects of MWCNTs, coexist within the ceramic body, significantly enhancing the mechanical properties of the CZP ceramic.

As the amounts of ZrO_2_ and MWCNTs added to the ceramic increase, numerous pores begin to form on the surface. This phenomenon leads to a marked reduction in tearing traces, while the ZrO_2_ particles grow larger and dispersibility declines, as demonstrated in Figure 7f,i. Excessive amounts of ZrO_2_ and MWCNTs tend to agglomerate, which disrupts the bonding between the grains of the ceramic matrix. As a result, during the growth of CZP grains, insufficient interfacial bonding between the grains leads to the formation of pores. The presence of pores affects the densification and inter-granular bonding within the ceramic, significantly reducing the sintering activity and resulting in a decline in its mechanical properties.

Thermal expansion tests were conducted on CZP ceramics after the composite toughening agent ZrO_2_/MWCNTs. The results indicated that 1.0Z/0.3M/C exhibited an average thermal expansion coefficient (TEC) of 2.54 × 10^−6^/°C in the temperature range from 100 to 700 °C. This value was significantly higher than that of conventional CZP ceramics, which had a TEC of 0.88 × 10^−6^/°C. According to the relevant literature, microcracks within CZP ceramics play a crucial role in their low thermal expansion properties. When CZP ceramics are heated and expand, these microcracks can relieve some of the deformation caused by thermal expansion, thereby helping to maintain their volume stability. However, following the composite toughening treatment, the propagation of microcracks in the ZrO_2_/MWCNTs/CZP ceramics was limited, which altered the original low thermal expansion characteristics of the CZP ceramics.

#### 3.2.4. Toughening Mechanism

**Figure 8:**
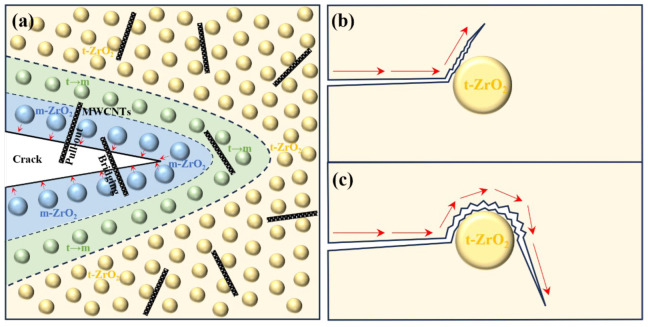
Toughening mechanism of ZrO_2_/MWCNTs/CaZr_4_(PO_4_)_6_ ceramics: (**a**) ZrO_2_ phase transformation toughening, and MWCNTs pull-out and bridging, and (**b**,**c**) crack propagation direction.

Firstly, during the high-temperature calcination process, the m-ZrO_2_ on the surface of the ceramic gradually transforms into t-ZrO_2_. The ZrO_2_ closely bonded to the ceramic matrix is constrained, preventing it from reverting to m-ZrO_2_ during the cooling process. The t-ZrO_2_ can be retained at lower temperatures, as described in Figure 8a. Secondly, when the ceramic is subjected to external forces that generate stress, this can induce the transformation of t-ZrO_2_ to m-ZrO_2_ while simultaneously forming numerous micro-cracks. This process relaxes the constraints imposed by the ceramic matrix on t-ZrO_2_. The volume expansion resulting from this phase transformation generates compressive stress (the red arrows in Figure 8a) at the crack tip, inhibiting further crack propagation. Meanwhile, the micro-cracks consume the energy for crack propagation, causing the cracks to deflect and branch during propagation. This effectively increases the length of the crack propagation path, as shown in Figure 8b,c. Eventually, the phase transformation leads to toughening of the ZrO_2_, enhancing the mechanical strength of the CZP ceramic material. Furthermore, the pull-out and bridging toughening mechanisms of MWCNTs are also present within the CZP ceramic matrix. Through the consumption of interfacial energy, the transfer and dispersion of stress further prevent crack propagation and induce crack deflection. In conclusion, the ZrO_2_/MWCNTs composite toughening agent within CZP ceramics exhibits multiple toughening mechanisms. The synergistic interaction among these mechanisms significantly enhances the mechanical properties of CZP ceramics.

## 4. Conclusions

This study aimed to explore the effects of ZrO_2_ and MWCNTs as single-phase and composite toughening agents on the mechanical properties and toughening mechanisms of CZP ceramics. The results are as follows:

1. When ZrO_2_ was used as a single-phase toughening agent, the optimal additive amount was found to be 10 wt.%, which resulted in the maximum relative density of Z/C ceramics. The flexural strength of the ceramics reached 71.60 MPa, representing a 137% increase compared to pure-phase CZP. Analysis indicated that the toughening mechanism is primarily attributable to the particle toughening effect of ZrO_2_.
2. The incorporation of ZrO_2_/MWCNTs as a composite toughening agent resulted in the ceramic sample 1.0Z/0.3M/C exhibiting remarkable mechanical properties. Specifically, the sample achieved a flexural strength of 138.43 MPa and a flexural modulus of 21.25 GPa.
3. A comprehensive examination of the toughening mechanism of the composite toughening agent revealed that the phase-transformation toughening mechanism of ZrO_2_—including the transformation between m-ZrO_2_ and t-ZrO_2_, the counteraction of compressive stress through phase transformation to prevent crack propagation, and the generation of microcracks that consume energy and cause crack deflection—interacted with the bridging and pull-out toughening mechanisms of MWCNTs. This interaction significantly enhanced the mechanical properties of CZP. However, excessive addition of agents can lead to agglomeration of the toughening materials, greatly reducing the sintering activity and the mechanical strength of CZP ceramics.

The toughening of CZP ceramics makes it one of the potential choices for thermal-shock-resistant ceramic materials in the aerospace field. In the future, research on toughening NZP ceramics can focus on applying further different toughening mechanisms to NZP ceramics to further enhance their mechanical properties and expand their application fields.

## Figures and Tables

**Table 1:** Experiment reagents.

Reagents	Purity	Manufacturer
CaCO_3_	99.00%	Tianjin Damao Chemicals Reagent Factory, Tianjin, China
ZrOCl_2_·8H_2_O	99.00%	Shanghai Aladdin Biochemical Technology Co., Ltd., Shanghai, China
(NH_4_)_2_HPO_4_	99.00%	Shanghai Aladdin Biochemical Technology Co., Ltd., Shanghai, China
ZrO_2_ (diameter ≤ 100 nm)	99.99%	Shanghai Aladdin Biochemical Technology Co., Ltd., Shanghai, China
MWCNTs (length 3–12 µm diameter 8–15 nm)	99.00%	Suzhou Tanfeng Technology Inc., Suzhou, China

**Table 2:** The effect of single-phase toughening of ZrO_2_.

Sample	Relative Density (%)	Flexural Strength (MPa)	Flexural Modulus (GPa)
CZP	-	57.47	14.00
1 wt.% Z/C	96.94 ***	62.91 ***	15.46 ***
5 wt.% Z/C	97.61 ***	67.97 ***	16.15 ***
10 wt.% Z/C	98.43 ***	71.60 ***	17.86 ***
15 wt.% Z/C	97.24 ***	66.59 ***	15.81 ***

Notes: The significance levels of 1% are denoted by ***. Please refer to the Supplementary Materials for the standard deviation of the data.

**Table 3:** The relative density, flexural strength, and flexural modulus of ZrO_2_/MWCNTs/CaZr_4_(PO_4_)_6_ ceramic samples.

Sample	Relative Density (%)	Flexural Strength (MPa)	Flexural Modulus (GPa)
CZP	-	57.47	14.00
1.0Z/0.1M/C	84.21 ***	107.45 ***	20.30 ***
1.0Z/0.3M/C	88.34 ***	138.43 ***	21.25 ***
1.0Z/0.5M/C	91.51 ***	123.03 ***	21.01 ***
1.0Z/0.7M/C	61.27 ***	56.21 ***	8.53 ***
3.0Z/0.5M/C	92.26 ***	111.95 ***	20.50 ***
5.0Z/0.5M/C	94.64 ***	108.53 ***	20.30 ***
7.0Z/0.5M/C	96.52 ***	109.91 ***	20.25 ***

Notes: The significance levels of 1% are denoted by ***. Please refer to the Supplementary Materials for the standard deviation of the data.

## Data Availability

The original contributions presented in this study are included in the article. Further inquiries can be directed to the corresponding authors.

## Supplementary Materials

The following supporting information can be downloaded at: https://www.mdpi.com/article/10.3390/ma18102289/s1, Table S1: Standard deviation of ZrO_2_/CaZr_4_(PO_4_)_6_ ceramic samples; Table S2: Standard deviation of ZrO_2_/MWCNTs/CaZr_4_(PO_4_)_6_ ceramic samples.
