# Supplementary material for: Synergistic Toughening Mechanisms in ZrO2/Multi-Walled Carbon Nanotubes-Reinforced CaZr4(PO4)6 Ceramics for Enhanced Mechanical Performance
Source: Materials (Basel). 2025 May 14;18(10):2289. doi: 10.3390/ma18102289 (PMC12113068; doi:10.3390/ma18102289)
Supplement: Supplementary file 1 [file materials-18-02289-s001.zip › materials-3570140-supplementary.pdf]

Table S1. Standard deviation of $ZrO_2/CaZr_4(PO_4)_6$ ceramic samples.

Sample	Relative Density (%)	S	Flexural Strength (MPa)	S	Flexural Modulus (GPa)	S
CZP	-	-	57.47	3.48	14.00	0.62
1 wt.% Z/C	96.94	0.23	62.91	2.82	15.46	0.57
5 wt.% Z/C	97.61	0.33	67.97	3.02	16.15	0.60
10 wt.% Z/C	98.43	0.25	71.60	2.49	17.86	0.49
15 wt.% Z/C	97.24	0.21	66.59	3.16	15.81	0.55

Table S2. Standard deviation of ZrO_2/MWCNTs/$CaZr_4(PO_4)_6$ ceramic samples.

Sample	Relative Density (%)	S	Flexural Strength (MPa)	S	Flexural Modulus (GPa)	S
CZP	-	-	57.47	3.48	14.00	0.62
1.0Z/0.1M/C	84.21	0.31	107.45	3.07	20.30	0.43
1.0Z/0.3M/C	88.34	0.24	138.43	2.55	21.25	0.55
1.0Z/0.5M/C	91.51	0.32	123.03	2.12	21.01	0.39
1.0Z/0.7M/C	61.27	0.31	56.21	2.86	8.53	0.21
3.0Z/0.5M/C	92.26	0.26	111.95	3.11	20.50	0.68
5.0Z/0.5M/C	94.64	0.29	108.53	2.35	20.30	0.72
7.0Z/0.5M/C	96.52	0.22	109.91	2.69	20.25	0.49
